# Mechanisms of traumatic cardiac arrest in Kuwait: A retrospective study

**DOI:** 10.1097/MD.0000000000020520

**Published:** 2020-06-05

**Authors:** Dalal Alhasan, Ameen Yaseen

**Affiliations:** a Department of Applied Medical Sciences, Public Authority of Applied Education and Training, Health Sciences College; b Audit Department, Emergency Medicals Services, State of Kuwait.

**Keywords:** emergency medical services, mechanism of injury, traumatic cardiac arrest

## Abstract

The objective of this study is to describe the epidemiology and causes of traumatic cardiac arrest (TCA) in Kuwait aiming to provide a preliminary background to update the current guidelines and improve patients’ management.

This is a retrospective analysis of TCA cases retrieved from emergency medical services archived data between 1 January and 31 December 2017. The TCA cases were sub-grouped based on mechanism of injury then compared in terms of patient demographics, vital signs, patterns of injuries, resuscitation practices, and outcomes.

Outcomes; On scene mortality rate and pre-hospital return of spontaneous circulation.

Among the 204 TCA patients, 140 patients met the inclusion criteria. This whole group was then divided in to 4 subgroups: road traffic accident (RTA) 76% (n=106), fall from height (FFH) 13% (n = 18), slip/fall 4% (n = 6), and assaults 7% (n = 10). There was significant difference between the four mechanisms in: mean age (*P* *=*  < .001), type of injury (*P* *=* .005), head injury (*P* *=* .005), chest injury (*P* *=* .003), GCS score < 9 (*P* *=* .004) and initial hypertension (*P* *=*  < .001). Initial hypertension and GCS score < 9 were only documented in head injuries of RTA and slip/fall groups. Significant difference was also seen in cardiopulmonary resuscitation (*P* *=* .006), airway management (*P* *=* .035) and on scene mortality rate (*P* *=* .003). All patients who had isolated head injury in FFH were pronounced dead on scene, 60%.

Not all TCA incidents are the same, there are different pattern of injuries in each TCA mechanism. Head injuries are predominantly seen in RTA, FFH, slip /falls and chest injuries are seen in assaults. This can influence emergency medical services personals resuscitation plan. Further research is required to address the resuscitation of TCA of different mechanisms.

## 1 Introduction

Unintentional injury is the leading cause of mortality and morbidity in young adults worldwide.^[1]^ Traumatic cardiac arrest (TCA) alone causes 5.1 million deaths per year.^[1]^ TCA is defined as cardiac arrest resulting from an external application of kinetic energy.^[2]^ And while the National Association of Emergency Medical Services Physicians (NAEMSP), the American College of Surgeons Committee and the Western Trauma Association proposed some guidelines for TCA management in the pre-hospital setting, the complex pre-hospital setting and TCA variable presentations made TCA management very challenging.^[3–4]^ Understanding common mechanisms of TCA incidents can help in predicting pattern of injures, adapting effective interventions, and hopefully improves TCA outcomes. One large scale study in England and Wales emergency departments on TCA identified head injury as the commonest injury pattern in TCA.^[5]^ The study recommended further research to evaluate and treat reversable underlying injuries of TCA.^[5]^ To our knowledge, there are limited number of studies on TCA mechanisms in the pre-hospital setting.^[2,6]^ This study is designed to describe the epidemiology and causes of TCA in Kuwait aiming to provide a preliminary background to update the current guidelines and improve patients’ management.

## 2 Method

### 2.1 Setting

Kuwait has a single, centralized dispatch center for all ambulance services; this is Arabic-based system and receives calls from the public and inter-hospital transportation. For emergency calls, Kuwait follows a European emergency response system. A universal emergency number 1–1–2 has automatic location identification with centralized dispatch for police, fire, and emergency medical services (EMS). If medical assistance is needed, the call is forwarded to an EMS call-taker who answers the call, reconfirms the address and responds by activating the nearest ambulance. The dispatched ambulance is staffed with 2 Emergency Medical Technicians (EMT) or 1 paramedic and 1 EMT. EMTs provide basic life support and paramedics provide advanced life support. Both based on North American resuscitation guidelines. As for TCA management, Kuwait EMS follows NAEMSP recommendation on TCA “if a patient presents or develops a blunt traumatic arrest, initiate cardiopulmonary resuscitation (CPR) on scene”.^[3]^ The local EMS protocol restricts scene time to 10 minutes and the current local EMS response time is 9.3 ± 5 minutes.^[7]^

This study was a retrospective analysis of TCA cases retrieved from EMS archived data between 1 January and 31 December 2017. The TCA cases were then sub-grouped based on mechanism of injury and compared in terms of; patient demographics, patterns of injuries, vital signs, EMS personal resuscitation, and outcomes. Primary outcomes were; on scene mortality rate and pre-hospital return of spontaneous circulation (ROSC).

### 2.2 Participants

Study population included adult patients (>18 years old) with TCA that activated EMS and were treated and transported by EMS. TCA cases caused by road traffic accident (RTA), fall from height (FFH), slip/fall and assaults were included in the analysis. Patients with cardiac arrest aetiology, patients for whom resuscitation was not attempted (decapitation, rigor mortis and dependent lividity) were excluded.

### 2.3 Data collection/measurement

Patient report forms were the only data source for TCA cases. Patient report forms are filled on scene by EMS personal and then stored in EMS audit department archived files. The researcher collected manually patient report forms from EMS audit department archived data. All data were present in the patient report form including patient demographic, mechanism of injury, final diagnosis, patterns of injury, vital signs, resuscitation practices, and outcomes.

Patient report forms with cardiac arrest as a final diagnosis and mechanism of injury of RTA, FFH, slip/fall and assaults were included in the analysis. Pattern of injuries were based on the anatomical location of the injury with special attention to head injuries. Because of England and Wales large scale study results.^[5]^ The investigator decided to analyze head injuries further. Head injuries data were collected then subdivided to isolated head injury and head injury associated with other injuries.

As for vital signs, those recorded by EMS personals’ on initial patient assessment were included. Pulse and respiratory rate were reported as recordable and non-recordable. GCS and BP were documented as; GCS score < 9 and initial hypertension (systolic blood pressure>140 mm Hg and diastolic Blood pressure > 90 mm Hg). This is because GCS score < 9 indicates severe traumatic brain injury.^[8]^ And initial blood pressure raise is common in severe head injury.^[9]^ Initial hypertension is the first stage of the Cushing reflex (a sympathetic response to head injuries with increases in intracranial pressure).^[9]^

Resuscitation practices included: CPR, airway management, and intravenous fluid resuscitation. Lastly, TCA outcomes were on scene mortality rate and pre-hospital ROSC.

During the project, all data were kept in a password locked computer files that only the research investigator can open. No data sharing was allowed outside the context of this project.

### 2.4 Sample size

Convenient sampling was used in this study. All eligible TCA patients treated by EMS during the study period were included.

### 2.5 Statistical methods

Statistical analysis was performed using Excel and Statistical Package for Social Sciences (IBM SPSS Version 23, NY). TCA mechanisms subgroups were compared using Chi-squared test for dichotomous variables, and Student's *t*-test for continuous variables. Two-sided tests were applied and a *P*-value ≤.05 was interpreted as statistically significant. Missing data were kept missing, i.e., not imputed or estimated.

### 2.6 Ethical considerations

The study had IRB approval from Kuwait Ministry of Health Independent Ethics Committee on 26 August 2016 (No.448). No informed consent was sought from participants. This is because all data were kept anonymous during this research.

## 3 Results

**Figure 1:**
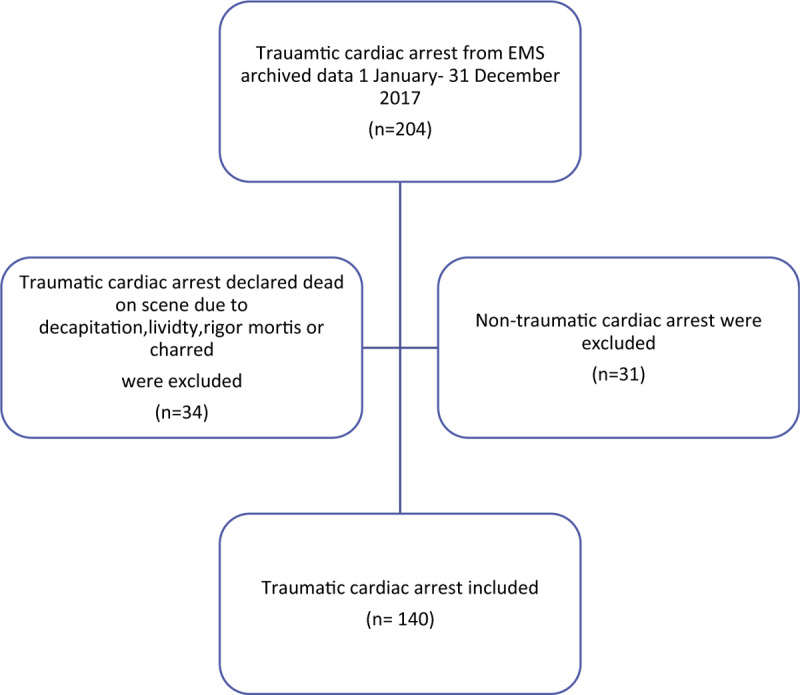
Flow chart of study population.

Out of the 204 TCA patients, 140 patients met the inclusion criteria (Fig. 1). Patients were divided into 4 subgroups: RTA 76% (n = 106), FFH 13% (n = 18), slip/fall 4% (n = 6), and assaults 7% (n = 10). Patients’ demographics, vital signs, patterns of injury, resuscitation, and outcome were compared between the 4 groups (Tables 1 and 2).

**Table 1:**
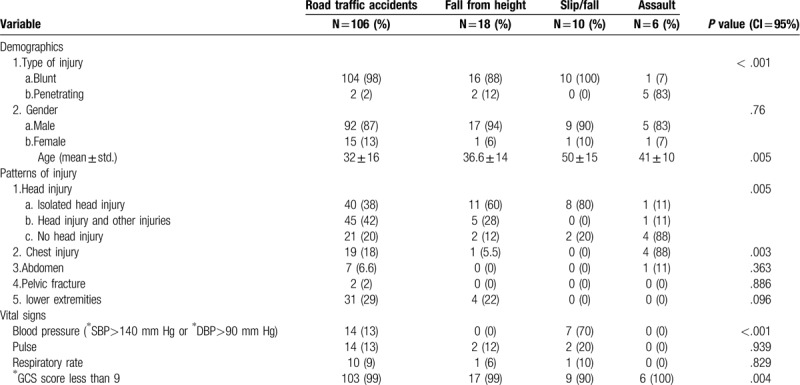
A comparison between traumatic cardiac arrest mechanisms in terms of patients’ demographics, vital signs, and patterns of injury, using Chi-squared test.

**Table 2:**
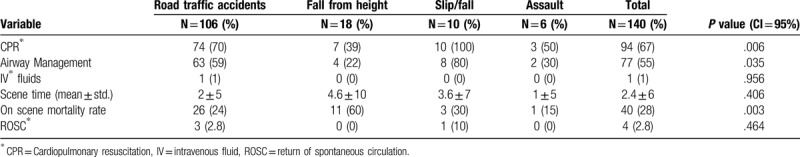
A comparison between traumatic cardiac arrest mechanisms in terms of resuscitation and outcomes, using Chi-squared test.

Our results showed significant difference in the mean age (*P* *=* < .001), type of injury (*P* *=* .005), head injury (*P* *=* .005), and chest injury (*P* *=* *.003)*,) between the 4 groups. GCS score < 9 and initial hypertension were only documented in head injuries of the RTA and slip/fall subgroups, and showed significant difference with *P* *=* .004 and *P* *=* <.001 respectively (Table 1).

Our results also showed significant difference in CPR (*P* *=* .006), airway management (*P* *=* .035) and on scene mortality (*P* *=* .003). All patients who had isolated head injury in FFH group (60%) were pronounced dead on scene (Table 2).

## 4 Discussion

This study described the different etiologies of TCA in Kuwait, which is not reported before. Our results showed that FFH had the worst TCA outcome. The on scene morality rate was equal to 60% (*P* *=* .003). The current study FFH mortality rate is higher than other studies, 2.7%.^[10]^ In terms of ROSC, there was no significant difference between the 4 subgroups.

In demographic characteristics, there was significant difference in age across the 4 subgroups (*P* *=* .005). RTA patients were young adults, mean age of 32 ± 16 and fall/slip patients were middle aged adults, mean age 50 ± 15. These findings are partially consistent with the present literature.^[1]^ young adults were seen in RTA,^[1]^ whereas elderly patients were reported in fall/slip.^[11]^

In terms of TCA patterns of injury, head injury was widely seen in RTA, FFH, slip/fall, and chest injuries were seen in assaults. This adds to England and Wales study results on TCA, the study identified head injury as the commonest injury pattern in TCA patients.^[5]^ However, it did not identify head injury subtypes or associate head injury with any mechanism of injury.

In RTA, 80% of the patients had head injury and approximately half of these patients had other injuries (Table 1), these findings are different from regional studies, Bener et al (1992) reported head injury was seen in 40% of RTA patients and only 14% had other injuries.^[12]^ One more important finding for RTA patients with head injury was the unstable physiological parameters. Our study observed initial hypertension and GCS score < 9 only in RTA patients with head injury. The documentation of those unstable parameters in RTA head injuries is vital. Grady et al (1988) declared hypertension and low GCS indicate raised intracranial pressure.^[9]^ Head injuries with raised intracranial pressure have specific pre-hospital management and transportation guidelines.^[13–14]^ Our study shed a new light on the physiological parameters of RTA patient's with head injury. Further investigations by other cohorts or randomized controlled trials are needed to confirm these associations.

We also showed FFH led to lethal isolated head injury, 60% (*P* *=* .003) and only few FFH patients had chest injuries of 5.5% (*P* *=* .003) (Table 1). This is different from the current literature, 88% of FFH patients had head injuries however 15% of them were lethal.^[15]^ Chest injuries were seen more frequently in FFH, 28%.^[15]^

Again the majority of slip/fall incidents had isolated head injuries, 80%. This is inconsistent with the literature, head injuries were seen in 10% of United States slip/fall incidents.^[16]^ Furthermore, this research showed not all slip/fall patients had high BP. Initial hypertension was only seen in head injury patients. This again can help EMS personal clinical and transportation decisions during slip/fall.^[13]^

In terms of slip/fall mortality, third of slip/fall patients were declared dead on scene.

In assaults, penetrating chest injury was the underlying cause of TCA. And they were the least to be declared dead on scene, 15%.

As for TCA resuscitation, CPR is recommended by the current NAEMSP guidelines^[3–4]^ Kuwait EMS implement these guidelines.^[7]^ And this research confirmed that the local TCA resuscitation format was CPR and airway management especially during RTA and slip/fall incidents (Table 2). However, adapting CPR by EMS personals deviated their focus from airway management, 55% (n = 77) and fluid resuscitation, 1% (n = 1) (Table 2). Airway management and fluid resuscitation are essential for treating TCAs with underlying etiology of head injury or chest injury. Furthermore, CPR impact on TCA outcome is controversial in the literature.^[17]^ This study again question CPR effectiveness during TCA of different mechanisms.

In this study we have established the dominant injury pattern in different TCA mechanism. Our results agree with the England and Wales report on head injury as the underlying cause for most TCA cases. We also confirmed that blunt trauma is frequent in RTA, FFH, slip/falls and penetrating injuries in assaults. These results highlighted the need of changing TCA resuscitation guidelines as CPR benefits appear questionable in certain situation. To our knowledge, this is the first study to report TCA mechanisms and patterns of injury in Kuwait.

This study had some limitations. First, the study sample size is relatively small, However, it is important to mention that it is within range of previous studies sample sizes on pre-hospital TCA 89 participants^[18]^ to 1030 participants.^[19]^ Second, missing cases were excluded from the analysis, this can introduce reporting bias. Third, the researchers did not include witness rate as a variable. Witnessed TCA victims have better outcome.^[6]^ The reason for not including witness rate in the analysis is that it was not reported in EMS patient report forms. Fourth, the sample was nationally representative of Kuwait but might be not transferable to other countries. Most countries are heavily populated.^[20]^ One more limitation, although the study highlighted CPR is suboptimal and other ways should be identified to resuscitate TCA patients, further research is required to establish CPR impact on different TCA outcome.

## 5 Conclusion

Not all TCA incidents are the same, there are different pattern of injuries in each TCA mechanism. Head injuries are predominantly seen in RTA, FFH, slip /falls, and chest injuries are seen in assaults. This can influence EMS personals resuscitation plan. Further research is required to address resuscitation in TCA of different etiologies.

## Acknowledgments

The author would like to acknowledge and thank Dr Faisal Al Ghanim for his enormous help and support rendered in the course of gathering the necessary data for the study.

## Author contributions

Study conception and design: DA.

Acquisition of data: AY.

Analysis and interpretation of data: DA.

Drafting of manuscript: DA.

Critical revision: DA.

All authors read and approved the final manuscript.
